# Implicit emotion regulation deficits in individuals with high schizotypal traits: an ERP study

**DOI:** 10.1038/s41598-020-60787-9

**Published:** 2020-03-03

**Authors:** Delhii Hoid, Dong-ni Pan, Yi Wang, Xuebing Li

**Affiliations:** 1 Key laboratory of Mental Health, Institute of Psychology, Chinese Academy of Sciences, Beijing, 100101 China; 2 Department of Psychology, University of Chinese Academy of Sciences, Beijing, 10049 China

**Keywords:** Emotion, Emotion, Human behaviour, Human behaviour

## Abstract

Schizotypy is associated with poor emotion regulation that is thought to contribute to the development of psychotic symptoms and to indicate a predisposition to schizophrenia. Having focused primarily on the relationship between schizotypy and explicit emotion regulation, existing studies have, until now, neglected to acknowledge the potentially important role of implicit emotion regulation. Our aim in the current study was to investigate implicit emotion regulation deficits in schizotypy. To this end, we used a newly developed Priming-Identification (PI) ERP paradigm, consisting of a priming phase and an emotion identification phase, to test 30 individuals with schizotypy and 30 healthy controls while also acquiring EEG data. During the priming phase, we aimed to manipulate emotion regulation goals (i.e., to bring about an intended emotional state) by presenting a category of words related to emotion regulation alongside a category of control words. Associated brain responses occurring during the subsequent stage were indexed according to three ERP components: N170, early posterior negativity (EPN) and late positive potential (LPP). Results showed that, in the control group, priming words associated with emotion regulation led to enhancements in the early N170 amplitude and the middle EPN during expression identification. The same pattern was not observed in the schizotypy group. In summary, our results suggest the presence of deficits in the early and middle stages of the implicit emotion regulation process among individuals with high schizotypal traits.

## Introduction

The ability to regulate emotions, for example by reducing the experience of negative emotions and coping with stress, is of great importance to functioning in daily life^1^. Indeed, multiple mental disorders, particularly schizophrenia spectrum disorders, are closely correlated with emotion dysregulation or maladaptive emotion regulation strategies^2–4^. Strauss *et al*.^5^ found that patients with schizophrenia fail to down-regulate emotional responses, which may in turn contribute to increased negative emotionality. It has also been found that patients with schizophrenia tend to overuse suppression as an emotion regulation strategy, which may lead to a subsequent maladaptive reappraisal of emotion^6^. Such emotion regulation abnormalities and emotion processing deficits have also been observed in schizotypy, which forms part of a spectrum of schizophrenia disorders^7^.

Schizotypy refers to a widespread pattern of social and interpersonal deficits that share similar symptoms with those of schizophrenia^7^. Schizotypy is also considered a predictor of future schizophrenia^8^. A variety of forms of emotion regulation abnormalities and deficits have been observed in individuals with high schizotypal traits^8^. By requiring participants to use different emotion regulation strategies while watching humorous film clips, Henry *et al*.^9^ observed that participants with schizotypy found it more difficult to express their positive emotions. They also found that the extent to which individuals demonstrate the three dimensions of schizotypy symptoms (i.e. negative symptom, positive symptom, disorganized symptom) was positively correlated with the use of suppression as a coping strategy. Thus suggesting that the overuse of such a strategy may contribute to some behavioral deficits of blunted emotional experience. Kerns *et al*.^10^ have argued that individuals with positive schizotypy tend to rely more on poor emotion regulation strategies. Such a conclusion was based on their finding that during emotion processing, compared with healthy control, those with positive schizotypy displayed an increase in attention to their emotion but in the meanwhile a decrease in the ability to identify the content thereof. In addition, brain-imaging studies have provided neural evidence of impairment during emotion regulation in individuals with high schizotypal traits. For example, when faced with laboratory induced psychological stress, participants with schizotypy who had scored high on physical anhedonia exhibited greater deactivation in brain areas related to emotion regulation^11^.

From the perspective of the dual-process framework^12^, all of the processes mentioned above require conscious effort and can thus be classified as forms of explicit emotion regulation. According to this theory, both explicit and implicit types of emotion regulation are necessary for well-being. Implicit emotion regulation refers to a less effortful, more automatic type of regulation process. Although most research to date has focused on the association between explicit emotion regulation processing and the schizophrenia spectrum disorder, little is known about the role of the implicit form of emotion regulation and the presence of potential deficits to this process. Schizotypy has been shown to be associated with poor emotion performance^8^. Moreover, issues with emotion regulation and stress contribute to the development and symptoms of psychosis such as odd beliefs^10^. By investigating the relationship between implicit emotion regulation and schizotypal traits we aim to provide a more nuanced understanding of the role of emotion processing in schizophrenia spectrum disorders.

In order to explore the neural mechanisms of implicit emotion regulation, an effective experimental paradigm is essential. Mauss *et al*.^13^ used a modified sentence unscrambling task to manipulate implicit emotion regulation. Such a task yielded results suggesting that when participants had been automatically primed with goals and concepts related to emotion control, they experienced less anger during subsequent emotionally provocative situations. In one study by Foti and Hajcak^14^, rather than being explicitly told to use reappraisal or to alter their negative beliefs about a stimulus, participants were given brief descriptions that would make the presentation of subsequent images either more or less negative. These results indicate that this kind of implicit manipulation can moderate negative emotion at both behavioral and neurological levels. Importantly, the design employed by Foti and Hajcak’s^14^ used the similar strategy of “implicit reappraisal”, which provides a way for participants to re-evaluate the emotional meaning of stimulus in a covert and subtle manner. Mocaiber *et al*.^15^ found that, while viewing affective pictures, indices of implicit emotion regulation (i.e. reaction time and LPP amplitude) could be modulated when participants used the “implicit reappraisal” strategy. In their study, this strategy was brought about through the use of technical reports containing information about whether stimulus material presented was fiction or fact. Zhang & Lu^16^ examined the time course of implicit emotion regulation by using a facial Go/Nogo task. They found that implicit emotion regulation modulates early ERP components and that Go-N2 and Nogo-P3 can signal implicit emotion regulation at the electrophysiological level. Despite the significant findings revealed by these implicit emotion regulation studies, the paradigms used are limited in a number of ways. Specifically, they are either not able to manipulate the goals of emotion regulation, that is, they concern only behavioral patterns without addressing neural mechanisms, or they investigate only late ERP components but not early and middle components.

As a response to these limitations, we adopted the recently developed Priming-Identification (PI) task to investigate potential deficits in implicit emotion regulation in a schizotypy group compared with a group of healthy control participants. This novel task contained a priming phase and an identification phase. A key aim of such a paradigm was to activate emotion-regulatory goals and produce the desired effects corresponding to these goals. The goal-driven nature of the task is typical of the aims of implicit emotion regulation, within laboratory settings at least^17^. Although the emotion-regulatory goals are well defined, it is paramount that they are activated implicitly, rather than be instructed directly. A series of five experiments in a study of Bargh *et al*.^18^ suggested that priming techniques could offer the advantage of activating nonconscious goals implicitly, with subsequent cognitive behaviors being guided by these goals. Mauss *et al*.^13^ also found that negative emotional responses could be reduced by priming emotion regulatory goals. By means of a word-matching task, the priming phase of our PI task was designed to prime two categories of words, namely emotion regulation-related (ER-related) words and emotion regulation-unrelated (ER-unrelated) words. We supposed that by priming ER-related words we could activate emotion-regulatory goals and enable implicit emotion regulation to function as desired. During the identification phase, participants were instructed to distinguish between two emotional faces, that we could investigate alterations in the emotional response process. In addition, we only presented threatening facial expressions (i.e. angry and fearful) in order to ensure that any alterations in emotional response were influenced solely by emotion regulation rather than emotion generation. Moreover, to clearly understand the mechanisms underlying implicit emotion regulation, we investigated the time course of processing using the technique of event-related potentials (ERPs), which is characterized by its high temporal resolution.

In light of previous studies that have investigated the temporal course of emotional facial processing^19–21^, we chose three ERP components as key indices in the current study. First, N170 is a negative peak at 150–180 ms occurring over occipital-temporal regions within the right hemisphere dominance^22,23^ that is very sensitive to human faces and is considered a key component in the structural encoding of faces^23,24^. Specifically, N170 is thought to be implicated in an early automatic modulation stage for the structural perception of facial stimuli^25^. Some researchers^26^ have, however, suggested that modulation of N170 in response to emotional faces is lacking in schizophrenia spectrum disorders. Thus, it could be argued that such deficits in this early stage of face processing could obstruct the more elaborative analysis of emotional information occurring in the next stage of processing, of which the second ERP component is implicated in. Specifically, the early posterior negativity (EPN) has been found to be the first cortical ERP component found to be selectively related to emotional processing of emotional stimuli^27^. This ERP component is considered as an informative index, typically starting around 200 ms or later after stimulus onset with a latency between 200–350 ms^28,29^. It is a bilateral temporal-occipital negativity that is responsive to emotional pictures versus neutral ones^30^. Third, the late positive potential (LPP) appears around 500 ms after stimulus presentation with a posterior midline scalp distribution. LPP is thought to reflect the allocation of attention to emotional stimuli that is necessary for motivation^31,32^. In previous studies, LPP was found to be modulated by different emotion regulation strategies. For instance, both reappraisal and suppression strategy could reduce LPP amplitudes^33^. Horan *et al*.^34^ provided electrophysiological evidence that patients with schizophrenia had an impairment in response to emotion regulatory manipulation, specifically, their LPP amplitudes showed no reduction under emotion regulation condition. Taking all of these considerations into account, we selected these three components as indices of our ERP results. Based on the latency of these three ERP components, the time course of emotion processing can be divided into three separate stages: an early stage (100–200 ms), a middle stage (200–300 ms), and a late stage (>400 ms). Consequently, the time course of emotion processing also can be represented by N170, EPN and LPP.

It has been demonstrated that, under non-explicit task conditions, the N170 can be influenced by emotional expression^35^. Following this, the EPN is related to more strategic decoding of emotional information in facial expressions^36^. Later, the LPP has been shown to index selective attention toward emotional stimuli^37^ and more elaborate processing^38^. As a consequence, it is reasonable to state that implicit emotion regulation of facial stimuli could also be indexed by these three components. Two studies have investigated the alteration of these three ERP components under different priming conditions using the newly developed PI paradigm. Wang & Li^39^ explored the time course of implicit emotion regulation in healthy people and observed a larger N170 amplitude induced by implicit emotion regulation, while EPN and LPP amplitudes were not influenced. This result led the authors to argue that the N170 could serve as an effective index of implicit emotion regulation. Liu *et al*.^40^ investigated implicit emotion regulation deficits in individuals with trait anxiety (i.e. high and low trait anxiety). Results indicated that individuals with high trait anxiety showed implicit emotion regulation deficits during the early and middle stages of emotion processing. That is, the enlargements in N170 and EPN amplitudes produced by individuals with low trait anxiety were absent, and no significant differences under two different priming conditions were produced.

Taken together, the aim of the current study was to investigate potential deficits in implicit emotion regulation in a schizotypy group compared with a group of healthy control participants. To this end, we applied the newly-developed ERP version of the PI paradigm and raised three hypotheses based on previous studies. First, at the behavioral level, negative emotion experience scores would be reduced by priming ER-related words in the control group, while such a reduction would be absent in the schizotypy group. We posed the hypothesis for this negative emotion rating in the schizotypy group based on their most prominent feature, “emotion paradox”, which is an apparent disjunction between external expression and internal emotion feelings^41^. We considered that, due to such a disjunction in schizotypy group, their negative experience scores would not reduce during implicit emotion regulation. Second, we hypothesized that, in the healthy control group, the early N170 amplitude and the middle EPN amplitude would be enlarged by ER-related priming, while the late LPP amplitude would not be affected. In other words, our results would be able to replicate the ERP results yielded by healthy control participants reported in a previous PI paradigm-based study by Liu *et al*.^40^. Third, since the previous words^26^ concluded that those falling within the schizophrenia spectrum disorder lack efficiency in decoding facial structure, which may thus further obstruct more elaborative processing for emotional information, we hypothesized that the schizotypy group would not show any significant differences in N170 and EPN amplitudes after being primed with two conditions. Previous research has reported that schizophrenia spectrum disorders show insensitivity to explicit emotion regulation, which is reflected by lack of modulation of LPP during emotion regulation process^34^. However, Wang and Li^39^ have pointed out that the late stage of implicit emotion regulation in the PI task, as indexed by LPP, was free of the cost of cognitive resources, and that there was no difference between LPP amplitudes under different priming conditions. Thus, we hypothesized that different priming conditions would not significantly alter LPP amplitudes in both stages. In summary, we hypothesized that individuals with high schizotypal traits would show implicit emotion regulation deficits in the early and middle stages of emotion process.

## Results

### Subjective ratings

**Figure 1:**
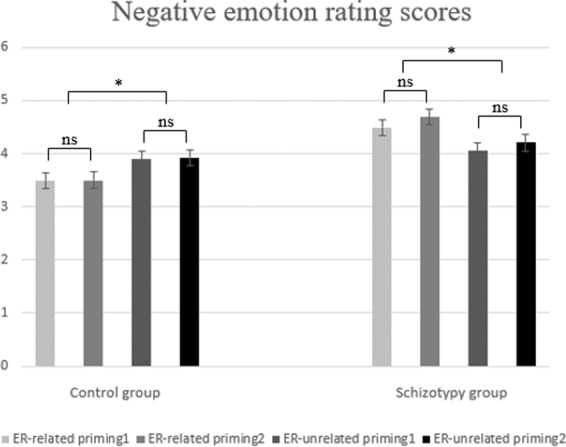
Bar plots for negative emotion rating scores. The larger rating scores indicated stronger negative emotion experience. “1” and “2” refer to order of rating. Error bars indicate the standard errors (* means *p* < 0.050).

Negative emotion rating scores were analyzed using a 2 (priming conditions: ER-related/ER-unrelated) × 2 (ordinal positions: first/second) × 2 (groups: schizotypy/control) repeated-measure ANOVA which revealed a significant interaction between priming condition and group (F (1, 58) = 9.817, p = 0.003, ηp^2^ = 0.145). A simple effects test revealed a significant main effect of priming condition for both the schizotypy group (F (1, 58) = 5.395, p = 0.024, ηp^2^ = 0.085) and the control group (F (1, 58) = 4.445, p = 0.039, ηp^2^ = 0.071). With regards to differences in ratings between the two groups, we observed that, for the schizotypy group, rating scores were higher after ER-related priming than after ER-unrelated priming, while, for the healthy control group, rating scores were higher after ER-unrelated priming than after ER-related (see Fig. 1). A simple effects test also showed that the difference between scores after ER-unrelated priming within the two group was not significant (p = 0.576), however, the difference between scores after ER-related priming within the two group was significant (F = (1,58) = 7.013, p = 0.010, ηp^2^ = 0.108).

### Reaction time

To investigate reaction time performance during the facial expression identification task, we conducted a 2 (priming conditions: ER-related/ER-unrelated) × 2 (group: schizotypy group/control group) ANOVA. Results indicated no significant main effects of priming condition (F (1, 58) = 0.123, p = 0.727) and group (F (1, 58) = 2.257, p = 0.138). There was also no significant interaction between priming condition and group (F (1, 58) = 1.376, p = 0.246).

### Intention detection

Based on their post-experiment verbal report, no participants indicated that the aim of the word-matching task was to regulate negative emotion in the following task.

### ERP Results

#### N170

**Figure 2:**
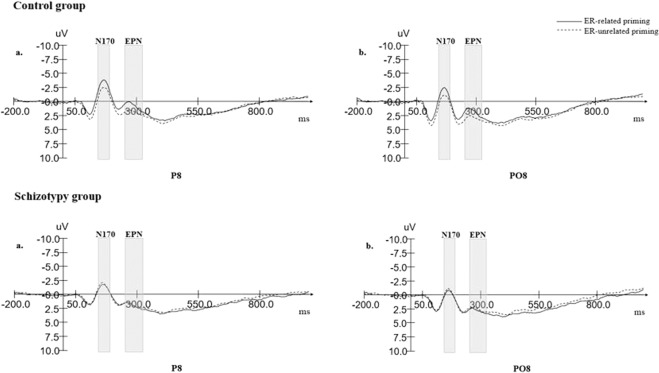
Grand averages of N170 and EPN amplitudes in control group and schizotypy group.

For N170 amplitudes, a 2 (priming conditions: ER-related/ER-unrelated) × 4 (electrode sites: P6/PO6/P8/PO8) × 2 (groups: schizotypy group/control group) repeated-measures ANOVA was conducted. We observed a significant interaction between condition and group (F (1, 58) = 18.608, p < 0.001, ηp^2^ = 0.243). A simple effects test revealed a significant main effect of priming condition in the control group (F (1,58) = 28.688, p < 0.001, ηp^2^ = 0.331), which was explained by the finding that the “ER-related” priming condition (M = −1.545, SD = 0.507) elicited more negative amplitudes than the “ER-unrelated” priming condition (M = −0.575, SD = 0.498). However, we did not observe such a main effect in the schizotypy group (F (1.58) = 0.554, p = 0.460) (see Fig. 2).

Results from three-factor-repeated-measure ANOVA also indicated a significant main effect of electrode sites (F (3, 56) = 17.725, p < 0.001, ηp^2^ = 0.487) whereby N170 amplitudes in P8 (M = −1.969, SD = 0.379) were significantly larger than those in the other electrode sites.

#### EPN

For EPN amplitudes, we conducted a 2 (priming conditions: ER-related/ER-unrelated) × 4 (electrode sites: P6/PO6/P8/PO8) × 2 (groups: schizotypy group/control group) repeated-measures ANOVA. Result indicated a significant interaction between condition and group (F (1, 58) = 12.905, p < 0.001, ηp^2^ = 0.182). A simple effects test showed a significant main effect of priming condition in the control group (F (1,58) = 17.606, p < 0.001, ηp^2^ = 0.233), whereby the “ER-related” priming condition (M = 2.144, SD = 0.547) elicited more negative amplitudes than the “ER-unrelated” priming condition (M = 3.045, SD = 0.556). We did not observe such a main effect in the schizotypy group (F (1.58) = 0.782, p = 0.380) (see Fig. 2).

Results from three-factor-repeated-measure ANOVA also showed a significant main effect of electrode sites (F (3, 56) = 16.881, p < 0.001, ηp^2^ = 0.475), whereby EPN amplitudes in P8 (M = 1.755, SD = 0.411) were significantly larger than those in the other electrode sites.

#### LPP in the early window (350–600 ms)

**Figure 3:**
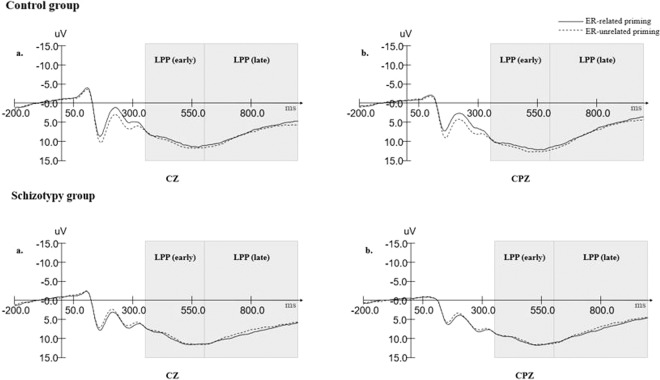
Grand averages of LPP amplitudes in control group and schizotypy group.

For LPP amplitudes during the early window, we conducted a 2 (priming conditions: ER-related/ER-unrelated) × 3 (electrode sites: CZ/CPZ/PZ) × 2 (groups: schizotypy group/control group) repeated-measure ANOVA. We observed no significant main effect of priming condition (F (1, 58) = 0.476, p = 0.493), group (F (1, 58) = 0.007, p = 0.936) nor any interaction between priming condition and group (F (1, 58) = 0.974, p = 0.328) (see Fig. 3).

Results also indicated that the main effect of electrode sites was significant (F (2, 57) = 3.518, p < 0.001, ηp^2^ = 0.241), such that LPP in CPZ yielded the largest amplitude (M = 10.982, SD = 0.635).

#### LPP in the late window (600–1000 ms)

For LPP amplitudes during the late window, a 2 (priming conditions: ER-related/ER-unrelated) × 3 (electrode sites: CZ/CPZ/PZ) × 2 (groups: schizotypy group/control group) repeated-measure ANOVA was conducted. We did not observe any significant main effect of priming condition (F (1, 58) = 0.292, p = 0.591), group (F (1, 58) = 0.661, p = 0.420) nor any interaction between priming condition and group (F (1, 58) = 1.843, p = 0.180) (see Fig. 3).

We observed that the main effect of electrode sites was significant (F (2, 57) = 16.525, p < 0.001, ηp^2^ = 0.222), such that LPP in CZ yielded the largest amplitudes (M = 8.411, SD = 0.566).

### Psychometric properties for ERP components

Reliability and dependability of N170, EPN, LPP were shown in Table 1.

**Table 1:** Psychometric properties for ERP Components.

Measure	N170		EPN		LPP (early)		LPP (late)	
	ER-related	ER-unrelated	ER-related	ER-unrelated	ER-related	ER-unrelated	ER-related	ER-unrelated
**Minimum Number of Trials**								
Schizotypy	31	31	26	22	15	9	53	39
Control	19	21	30	21	17	16	34	32
**Spilt-half Reliability**								
Schizotypy	0.91	0.97	0.84	0.94	0.97	0.98	0.89	0.89
Control	0.89	0.91	0.82	0.91	0.91	0.95	0.78	0.86
**Dependability (95% CI)**								
Schizotypy	0.93 (0.89, 0.96)	0.92 (0.87, 0.96)	0.91 (0.85, 0.95)	0.92 (0.87, 0.96)	0.94 (0.91, 0.97)	0.97 (0.95, 0.98)	0.83 (0.73, 0.91)	0.87 (0.79, 0.93)
Control	0.89 (0.83, 0.94)	0.89 (0.82, 0.94)	0.89 (0.83, 0.94)	0.92 (0.87, 0.96)	0.94 (0.90, 0.96)	0.94 (0.90, 0.97)	0.88 (0.80, 0.93)	0.89 (0.82, 0.94)

‘Minimum number of trials’ refers to minimum number of trials to reach the minimum dependability point estimate of ≥0.80.

## Discussion

The current study was conducted to investigate potential implicit emotion regulation deficits in a schizotypy group compared with a group of healthy control participants. First, we observed a priming effect at the behavioral level in the control group that was absent in the schizotypy group. Specifically, by reducing negative emotional experience, the ER-related priming condition benefitted healthy control participants but not those participants in the schizotypy group. Second, we found that ratings were not modulated as a function of when they were reported. In other words, the implicit effect of priming was not reduced with time. Third, we found that priming ER-related words produced an enlargement in the early N170 and the middle EPN amplitudes during the face expression identification task. However, this was only observed in the control group and was absent in the schizotypy group. For the late stage of emotion processing, LPP during both early and late time window were not modulated by priming words in either group. These results demonstrate the presence of processing deficits at the early and middle stages of implicit emotion regulation in individuals with high schizotypal traits.

The previous studies found that the schizophrenia spectrum disorder is along with subjective-objective disjunction refering to a paradoxical pattern between subjective reports and objective measures^42^. Such paradox could be observed in many functional domains, for instance, in neuropsychological functions and affective experience. Specifically, individuals with high schizotypal traits exaggerate the extent of their subjective concerns which is far greater than the level of objective measures^42^. Li *et al*.^43^ adopted a multidimensional approach to reveal that the deficit paradox not only exists in basic affective process, but also in emotion regulation. More specifically, schizotypy group reported notable disruptions in emotion regulation using self-reports, however, their performance in implicit emotion regulation task, an objective measure, did not differ from that of healthy control. In the current study, after activating emotion regulation goals by ER-related words, individuals from the schizotypy group rated their emotions more negatively compared to ratings after priming ER-unrelated words. If schizotypy group have deficits in implicit emotion regulation, referring to the performance of anxiety who showed similar deficits in subjective ratings after ER-related priming condition^40^, the scores under two priming conditions in schizotypy group should not differ significantly. Hereby, the more negative rating after ER-related priming of the schizotypy individuals in the current study may be caused by a pattern of paradox in emotion regulation process. Cohen *et al*.^42^ suggested that cognitive bias may underlie such subjective and objective disjunction to some extent. Thus, we speculated that even if ER-related priming could not modulate negative emotion of schizotypy, a potential cognitive bias may prompt schizotypy group to exaggerate their negative feelings. Li *et al*.^43^ also indicated that the experience of ambivalence for shcizotypy might inducing opposed emotional feelings.

Since emotion regulation deficits were present at the behavioral level in the schizotypy group, the neural mechanism underlying these deficits should be further discussed. Three related ERP components (i.e. N170, EPN and LPP) were selected as a means of investigating the time course of implicit emotion regulation in schizotypy. At the earliest stage of implicit emotion regulation, under the ER-related priming condition, we observed an enhanced N170 during the facial expression identification task in the control group that was absent in the schizotypy group. As has been well established, N170 is a face-specific ERP component^44^, but some debate exists over whether N170 is sensitive to facial expression. Some studies^45,46^ have shown that N170 only reflects structural encoding stages rather than the processing of affective valence. This was, however, not supported by a recent meta-analysis^47^ that suggested N170 is sensitive to facial expression. It should be noted that, despite their differences, previous studies mostly agree on the notion that N170 is related to the encoding of facial configuration. Thus, we speculate that N170 in implicit emotion regulation reflects encoding of facial structure. According to a model put forward by Bruce and Young^48^, the early stage of functional face recognition involves first perceiving a face, viewers then construct a viewer-centered description consisting of the layout of the surfaces which is followed by the image of the face it gives rise to. Bentin *et al*.^49^ suggested that N170 may reflect a component of the structural encoding stage. It is, therefore, possible that N170 in implicit emotion regulation is corresponding to viewer-centered descriptions, which provide information for the analysis of expression. During this process the configuration of various features leads to the categorization of emotional expression. Thus, we speculate that the enlargement of N170 in the early stage of implicit emotion regulation reflects, to some extent, facilitation in the process of decoding facial expression. An MRI study has shown that the volume of gray matter in the fusiform gyrus, a region implicated in the configural system reflected by the N170 component^50^, is smaller in patients with first-episode schizophrenia compared to controls^49^. In accordance with this viewpoint, another MRI study^51^ has also demonstrated that the fusiform gyrus subserves the encoding of faces, and that some neuroanatomic fusiform gyrus abnormalities exist in schizophrenia. Thus, we argue that the absence of any priming-derived enlargement in N170 in the schizotypy group is a result of such abnormalities in individuals falling under the schizophrenia spectrum personality disorder. Consistent with our own findings, Agustin *et al*.^52^ indicated that individuals with schizophrenia and related disorders carry deficits in decoding facial features. Such a deficit in schizotypy would obstruct elaborative analysis of emotional information at the subsequent stage in the time course of implicit emotion regulation. Moreover, this would exert consequent deficits on facial identification.

Regarding the post-priming alteration of EPN amplitude after priming, the present study shares some similarities with previous studies investigating trait anxiety^40^. While control groups in both studies produced enlargement of EPN amplitude during the facial expression identification task after ER-related priming, we observed an absence of such an alteration in both the schizotypy group and the anxiety group. In general, EPN is thought to reflect early selective visual processing of significant emotional information^53^. From the perspective of early and middle stages of overall emotion processing, it has been shown that during face processing, from the early time point to around 250 ms, an abundance of information has been processed in the human brain^54^. Considering that a component implicated in the early stage of face perception such as N170, is more related to encoding facial configuration^55^, EPN and its enlargement could be understood to be more sensitive to emotional information per se. Thus, we can speculate that an increase in EPN amplitude occurring over the time course of implicit emotion regulation in the healthy control group was related to a facilitated selection of emotional information thus leading to a more accurate identification of facial expression. Previous research has suggested a source for the EPN in the primary and secondary visual cortices^56^, and that excitatory projections from the amygdala to this area are strengthened in response to emotional compared to neutral stimuli, thus leading to enhanced activation of the visual cortex^57^. Moreover, given the observation that the amygdala is responsive to threatening faces (i.e. fearful and angry)^58^, the EPN likely reflects an excitatory effect of amygdala on the visual cortices^59^. Some studies have found that schizophrenia patients along with their first-degree relatives, as well as individuals with high schizotypal traits all display decreased activity in the visual cortex^60,61^. Thus, we speculate that the absence of any post-priming EPN enlargement within the schizotypy group could be explained by deficits in visual cortex activation. Deficits in analyzing emotional information and discriminating emotion were correlated with negative symptoms of schizophrenia and some eccentric behaviors. The presence of such implicit emotion regulation deficits is likely to trouble individuals with high schizotypal traits during social interaction and in turn induce extensive negative emotion^62^.

With regards to the LPP amplitude, consistent with our hypothesis, we observed no priming effects in either group for this late stage component. Previous studies suggest that implicit emotion regulation does not demand significantly more cognitive resources at the late stage of emotion processing^39,40^, a conclusion which is also supported by our own results. In the context of explicit emotion regulation however, LPP amplitude has shown some differences. For instance, when participants were asked to view unpleasant pictures and simultaneously use a reappraisal strategy, a substantial reduction of the LPP amplitude was observed^63^. Yuan *et al*.^64^ found that both expressive suppression and cognitive reappraisal decreased subjective negative emotion to a similar extent. However, suppression was found to dampen unpleasant emotion stimuli more rapidly, albeit at the cost of more cognitive resources. This pattern was embodied by the finding that reductions in LPP for negative stimuli appear earlier during suppression compared to during reappraisal. Adrian *et al*.^65^ have also suggested that modulations in LPP amplitude in response to negative images were sensitive to different emotion regulation strategies. As mentioned above, LPP could represent a kind of supraliminal and cognitive-costing regulation during explicit processing. Combined with the view that LPP reflects increased allocation of attentional resources to motivationally relevant stimuli^66^, we speculate that LPP involvement in implicit emotion regulation represents a subliminal and positive regulation process. With regards to potential deficits to the late stage of emotion regulation within schizophrenia patients, research into LPP amplitude has yielded some evidence. Horan *et al*.^34^ adopted a picture viewing task with three conditions, namely neutral or unpleasant pictures preceded by neutral or negative descriptions (i.e. preappraisal). Results showed that LPP was enlarged during the initial period and 600–1500 ms epochs. They suggested that ‘modulation of neural responses by a cognitive emotion regulation strategy appears to be impaired in schizophrenia during the first two seconds after exposure to unpleasant stimuli.’ Moreover, it was reported that people at risk for schizophrenia spectrum disorder such as social anhedonia also have abnormalities in emotion regulation processing, which is reflected by enhanced LPP amplitude when passively viewing negative pictures compared to controls^67^. The absence of any post-priming LPP modulation from either ER-related and ER-unrelated conditions across schizotypy and control groups, leads us to speculate that deficits displayed by individuals with schizotypal traits in the late stage of explicit emotion regulation are not mirrored by deficits at the same stage of the implicit regulation.

Taken together, our study provides the following insights into implicit emotion regulation. Firstly, during the early stages of regulation, N170 may reflect the configural encoding of emotional faces. Secondly, during the middle stage of regulation, EPN represents a sustained attention to emotional information. Finally, at the late stage, LPP could be understood as a subliminal motivationally-relevant process. With respect to the schizophrenia spectrum disorders, results suggest that individuals with schizotypy display deficits in the early and middle stage of implicit emotion regulation process.

The present study was subject to some limitations. First, the aim of the present study was to investigate general deficits in implicit emotion regulation in schizotypy and provide neural evidence for these deficits. However, according to previous research^68^, schizotypy can be classified according to positive and negative symptomology, and these dimensions are associated with different emotional abnormalities^41^. Our study was not able to provide further insight about the association between different symptoms and implicit emotion regulation. It is still unclear as to which dimension of schizotypy makes the greatest contribution to these deficits. Conversely, we also have no insight into how deficits in implicitly regulating negative emotion affect both sets of symptoms. Future research needs to examine more closely the links between the different dimensions of schizotypy and implicit emotion regulation over different time courses. Second, we did not use physiological indices, such as heart rate and mean arterial blood pressure, to measure cardiovascular responses while participants performed the experiment. Cardiovascular measures have been found to be a more effective and objective indicator of negative emotional experience than subjective scores^17^. Future studies could benefit from adopting more physiological indices to detect the effect of implicit emotion regulation.

Previous work suggests that schizotypy is associated with poor emotional performance and that impairment in emotion regulation contributes to the development of psychosis^8^. Given this association, early identification of emotion regulation issues followed by appropriate intervention would prove indispensable to the prevention and treatment of psychosis. Despite this, few intervention methods with the aim of strengthening such emotion-based abilities have been reported^69^. Future research on schizophrenia spectrum disorders and adaptive emotion regulation strategies with appropriate training programs are thus required urgently.

## Conclusion

Using a recently developed Priming-Identification (PI) ERP paradigm, we investigated implicit emotion regulation processing in individuals with high schizotypal traits. Results suggested that these individuals show deficits in the early and middle stage of implicit emotion regulation. We speculate that such deficits may contribute to the development of psychosis. We argue that a clearer understanding of implicit emotion regulation and its associated deficits in individuals with schizotypy would be instructive for future research and the attenuation of symptoms in related disorders.

## Method

### Participants

704 undergraduate students in Beijing participated in an Internet screening using the Chinese version of the Schizotypal Personality Questionnaire (SPQ)^70,71^. Students with SPQ scores in the top and bottom 20% of the distribution were allocated to the schizotypy group and the healthy control (HC) group respectively. Sample size was determined based on the following two considerations: (1) According to previous PI paradigm-based studies, a usual number of 25 participants in each group were used^39,40^. (2) Estimated with a cohen’s f of 0.34 according to effect size of previous PI paradigm-based study^40^, the required number of subjects was 16 per group to achieve a statistical power of 95%. Finally, a total of 60 students consisting of 30 individuals with high schizotypal traits and 30 healthy subjects were recruited.

Participants in each group were all right-handed and had normal or corrected-to-normal vision according to their oral report. They were also matched for age, gender and level of education. Demographic characteristic for the two groups is shown in Table 2. All participants were free of antipsychotic medications and had no history of psychiatric disorders. The ethics committee of the Institute of Psychology, Chinese Academy of Sciences approved the experimental protocol for this research which was carried out in accordance with the approved guidelines. Every participant provided written informed consent in advance of the experiment and received 100 RMB as compensation for their attendance.

**Table 2:** Demographic information of Participants [Mean (SD)].

	Schizotypy	Control	t/χ^2^	p-value
Age (years)	21.20 (2.70)	21.33 (2.47)	−0.21	0.842
Gender (n male)	19	17	0.278	0.792
Education (years)	14.37 (2.48)	15.07 (2.49)	−1.099	0.276
SPQ (score)	44.90 (5.32)	11.33 (4.35)	26.755	<0.001

### Stimuli

In the present study, we adopted the same paradigm and stimulus used by Liu *et al*.^39^ Experimental materials included priming words, and pictures of faces. First, priming words were classified as “ER-related” and “ER-unrelated”. Based on prior evaluations by another group of Chinese students, “ER-related” words have been proven to be effective in decreasing negative emotion and modulating emotion regulation at an implicit level. The “ER-unrelated” category contained priming words that have previously been shown to have no correlation with emotion regulation. Second, for the facial pictures in the identification task, we selected 40 angry faces and 40 fearful faces from the Chinese Facial Affective Picture System (CFAPS)^72^. Equal numbers of faces from both males and females were presented. In addition, we found no significant differences in arousal evoked by fearful faces and angry faces (t (78) = −0.940, p = 0.351). For more details regarding experimental materials, refer to the previous PI paradigm-based study^39,40^.

### Experiment design and procedure

The PI paradigm consisted of two tasks: a word-matching task and a facial expression identification task. The purpose of the word-matching task was to manipulate emotion regulation goals by matching the meaning of the words. The aim of the facial expression identification task was to induce negative emotion. Thus, we were able to observe effects of different priming conditions during this identification phase.

For the word-matching task, three priming words were presented at different positions on the screen. Participants were required to press one of two keys to choose the word that matched the meaning of the word presented at the top of the screen, with “O” and “P” for words on the bottom left and right site of the screen, respectively. The word-matching task contained two different conditions, namely “ER-related” priming and “ER-unrelated” priming. For the ER-related priming condition, one of the two words that needed to be matched belonged to the category of “ER-related” words and the remaining one was selected from the “ER-unrelated” words. In the ER-unrelated priming condition, all three words consisted of “ER-unrelated” words.

The facial expression identification task consisted of a series of subtasks. After a 200–500 ms fixation presentation at the center of the screen, a facial picture from a random category was presented for 1000 ms. During this period, participants were required to respond as quickly as possible by pressing the appropriate key on the keyboard. The “K” and “L” indicated angry faces and fearful faces, respectively. Appropriate options were balanced across subjects. After completing a fixed number of trials (30 trials before first subjective rating and 24 trial before second subjective rating) for the identification phase, participants were required to rate the extent of their negative emotion along a 9-point scale.

**Figure 4:**
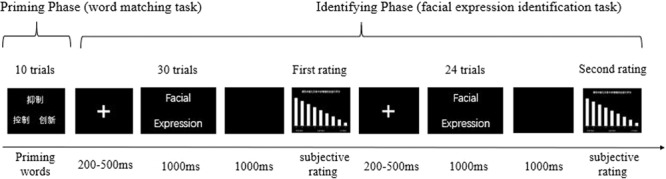
The specific trial procedure of Priming-Identification task.

The order of ER-related and ER-unrelated priming conditions was balanced between subjects. Both priming conditions contained three successive blocks. In each block, participants were asked to complete 10 trials for the word-matching task, followed by 54 trials for the facial expression identification task. During the facial expression identifying stage, participants were also asked to complete two ratings of their negative emotion (see Fig. 4 for Experimental Procedure). The facial expression identification task included 27 angry faces and 27 fearful faces. Therefore, the whole priming identification task consisted of 60 trials in the word-matching task, 324 trials in the facial expression task and 12 trials in the rating tasks. For the purposes of minimizing task-switching effects, the first six trials in each block of the facial identification task were excluded. Thus, a total of 360 trials were analyzed for every subject.

Upon completion of the priming identification task, participants performed an intention detecting test using self-report. This was used in order to rule out the possibility that participants were aware of the relationship between the priming task and the facial expression task. Based on the assumption that emotion regulation processes would not have occurred implicitly, those who detected the relationship between the two tasks would be excluded from final data analysis.

### EEG recording and processing

Continuous EEG was recorded during the facial expression identification task using a 64-channel Ag-AgCl electrode cap (NeuroScan Inc., Herndon, VA, USA) based on the 10–20 International System. All EEG data were recorded with an online reference to the left mastoid and analyzed with an offline re-reference to the mean of left and right mastoids. Two electrodes were placed above and below the left eye for recording vertical EOGs, and two electrodes were placed 1 cm outer from canthi for recording horizontal EOGs. Scalp impedance for each electrode was kept below 5 KΩ. Both EEG and EOG signal were filtered using a range of 0.05–100 Hz and sampled using the rate of 500 Hz.

During the offline analyzing period, EEG data were low pass filtered at 30 Hz (12 dB/oct). The ERP data were corrected using a baseline of 200 ms, and then segmented using a window of −200 ms to 1000 ms the face onset as the zero point in the expression identification task. Any trials whose EEG voltages exceeded ±80 μV were excluded from further analysis.

### Data analysis

We analyzed negative emotion ratings according to two different priming conditions and their order of rating (first and second) in the experimental procedure. We also calculated reaction times produced in the expression identification task that were associated with correct responses. We excluded trials with RTs less than 200 ms or more than 1000 ms. This left an average of 127 trials (90% of the total) under ER-related and ER-unrelated priming conditions for both healthy control and schizotypy groups that were ultimately analyzed.

For ERP data, during the facial expression-identifying phase, average amplitudes of correct trials in the two priming conditions were overlaid. Angry and fearful faces were both negatively valanced and, as mentioned above, did not yield any significant difference in arousal levels. In addition, the same priming procedure was conducted before displaying both kinds of faces. As a consequence of these three factors we combined amplitudes of angry and fearful faces for analyses. As mentioned above, any trials whose EEG voltages exceeded a threshold of ±80 μV during the recording phase were excluded from further analysis. Finally, an average of 120 trials (83% of the total) under the ER-related priming condition and 118 trials (82% of the total) under the ER-unrelated priming condition were analyzed from the healthy control group. An average of 122 trials (84% of the total) under the ER-related priming condition and 114 trials (79% of the total) under the ER-unrelated priming condition were analyzed from the schizotypy group.

Since early and middle ERP components in previous PI paradigm-based studies exhibited right hemisphere dominance, we analyzed N170 and EPN with a time window of 145–190 ms and 250–320 ms respectively in four electrode positions of the right hemisphere (P6, P8, PO6, PO8). For LPP, based on previous emotion regulation-related studies^33,66^ and morphology of the ERP waveforms in the present study, we inspected the LPP across two epochs: (1) an early window for LPP, 350–600 ms and (2) a late window for LPP, 600–1000 ms. We analyzed both time windows for LPP in three electrode positions (CZ, CPZ, PZ).

For psychometric properties, we analyzed split-half reliability using Spearman-Brown corrected formula to determine the reliability of N170, EPN, LPP. We used the ERP reliability Toolbox v. 0.4.5^73^ to estimate overall dependability of N170, EPN, LPP. All statistical analyses were conducted with SPSS 17.0 software with a significance level at 0.05. We used the Greenhouse-Geisser method to correct sphericity violations. Partial eta-squared (ηp^2^) were used to indicate effect sizes produced by ANOVA tests.
